# A double blind randomized controlled trial in neonates to determine the effect of vitamin A supplementation on immune responses: The Gambia protocol

**DOI:** 10.1186/1471-2431-14-92

**Published:** 2014-04-04

**Authors:** Suzanna LR McDonald, Mathilde Savy, Anthony JC Fulford, Lindsay Kendall, Katie L Flanagan, Andrew M Prentice

**Affiliations:** 1 Medical Research Council (MRC) International Nutrition Group (ING), London School of Hygiene & Tropical Medicine (LSHTM), Keppel Street, WC1E 7HT, United Kingdom & MRC Keneba, London, The Gambia; 2 Statistics, MRC Gambia Unit, PO Box 273, Banjul, The Gambia; 3 Vaccinology theme, MRC Gambia Unit, PO Box 273, Banjul, The Gambia; 4 Department of Immunology, Monash University, Melborne VIC 3181, Australia

**Keywords:** Neonatal, Vitamin A supplementation, Immune responses, Africa, Double blind randomised control trial

## Abstract

**Background:**

Vitamin A supplementation significantly reduces all-cause mortality when given between 6–59 months of age, but has a null or detrimental effect when given between 1–5 months. Studies of neonatal vitamin A supplementation conducted across Africa and South Asia have produced conflicting findings. These age-pattern variations might result from immunological interactions between vitamin A supplementation and vaccines. Knowledge on the potential immunological sequelae of human neonatal vitamin A supplementation is so scarce that the foremost aim of this study is to seek indicative data on aspects of immunity likely to be affected by neonatal vitamin A supplementation. The objective of this trial is to test whether human neonatal vitamin A supplementation modulates immune function including improved thymic maturation in infancy and improved systemic immune responses to routine immunization.

**Methods/design:**

In an area of moderate vitamin A deficiency in a peri-urban area of The Gambia, 200 mother–infant pairs were enrolled in a double-blind randomised controlled trial. Within 48 hours of birth, neonates were randomised with stratification by birth weight and sex to receive either an oral dose of 50,000 IU vitamin A or placebo. Expanded Programme of Immunisation birth vaccinations were administered after supplementation, with subsequent vaccinations administered at 8, 12 and 16 weeks of age. A range of immunological outcomes were examined up to 17 weeks of age, with additional morbidity and anthropometry follow-up carried out throughout the first year of life. The primary endpoint of this trial is the frequency of circulating T regulatory (T_reg_) cells expressing gut homing receptors in infants at 17 week post-supplementation, with secondary outcomes including thymus maturation and B cell immune responses.

**Discussion:**

Indicative immunological data from this trial (and its Bangladeshi counterpart), will complement the larger randomised controlled trials (conducted in India, Tanzania and Ghana), on the effectiveness and safety of neonatal vitamin A supplementation in improving infant survival. Combined these trials, in addition to the existing trials, will inform policy.

**Trial registration:**

clinicaltrials.gov NCT01476358

## Background

Within Africa and South-East Asia, vitamin A deficiency (VAD) (defined as a serum retinol concentration < 0.70 μmol/l), affects > 19 million pregnant women and 190 million children aged under 5 years [1]. This deficiency is a major contributor to eye disorders and mortality [2]. To reduce these risks, the world health organization (WHO) recommends administering periodic high-dose vitamin A (VA) supplements to children aged 6–59 months living in low-income countries, as at the time of policy formation this intervention had been shown to reduce all cause mortality by 23 to 30% in this age group [3]. However, while many younger infants are VAD, supplementation studies have shown no beneficial effects on child survival when given between 1–5 months [4]. Neonatal VA supplementation (NNVAS) has produced conflicting findings. Giving 50,000 IU VA to infants within the first month of life significantly reduced infant mortality in South Asian settings (Indonesia, India and Bangladesh) [5-7], but such beneficial effects have not been replicated in an African setting. Trials conducted in Guinea-Bissau and Zimbabwe have found no overall effect of vitamin A supplementation (VAS) given at, or just after birth, on infant survival [8,9]. A systematic review of the literature commissioned by WHO identified 6 randomized controlled trials (RCTs) involving 42,508 infants that evaluated NNVAS (given at < 1 month of age). A meta-analysis of these trials suggested no overall effect in infant mortality in the intervention group (pooled relative risk 0.92, 95% CI 0.75 to 1.12, P = 0.393; I^2^ = 54.1%, P = 0.053) [10]. The available evidence however is not enough to either accept or reject NNVAS as an intervention with considerable potential for improving infant survival. WHO therefore commissioned three new large trials of NNVAS in Ghana, Tanzania and India [11] with mortality as the primary outcome in conjunction with two smaller mechanistic immunological trials in Bangladesh and The Gambia. In conjunction with these trials, a study on VA metabolism in the piglet model was also commissioned.

Vitamin A plays an essential role the development of healthy immune responses [12]. Substantive evidence from animal (*in vivo* and *in vitro*) and human *in vitro* studies indicates that VA and its metabolites (particularly retinoic acid (RA)) have a powerful role in the regulation of both innate and adaptive immune responses [13,14]. In terms of innate immune responses, this includes the integrity of mucosal epithelia[15] and the numbers, differentiation and cytokine secretion profiles of monocytes, macrophages, natural killer cells and neutrophils [16,17]. With respect to adaptive immune response, it has been postulated that VA has a role in thymic development and maturation of thymocytes [18], therefore VAD may impair thymic function, with resultant effects on the peripheral T cell pool. Many studies have shown that VAS increases the number of T cells, particularly the CD4^+^ subpopulation and that it has a direct effect on cytokine production and T cell activation [17,19,20]. Animal studies have demonstrated that VA effects the helper T cell 1 (Th1)/Th2 balance, with VAD inducing a shift in the immune response towards Th1-cell-mediated immunity and VAS boosting Th2-type responses [21]. In addition, VAS suppresses Th17 responses and promotes regulatory T cell (T_reg)_ responses [22-24]; whilst inducing gut-homing markers (α4β7 and CCR9) in CD4^+^ and CD8^+^ T lymphocytes, T_reg_ cells and B cells[25-27]. Specific subsets of intestinal antigen-presenting cells present in the lamina propria, such as dendritic cells (DCs) and macrophages express RA synthesizing enzymes (aldh1a1 and aldh1a2), and therefore have the capacity to convert VA into RA (as reviewed in [28]). More recent data has shown that although RA plays an important role in the maintenance of intestinal tolerance and in immune homeostasis, during infection or autoimmune inflammation, it has the reciprocal role of promoting effector T cell responses[29]. RA has also been demonstrated to stimulate B cell maturation, activation and differentiation), and increase primary and memory antibody responses [30]. A very limited number of immunological studies have been conducted on VAS in human neonates. A RCT conducted in Guinea Bissau found no effect on NNVAS and immune responses to BCG vaccine at 6 months of age [31], however studies nested within this trial, analysed by sex, found that in boys less than 6 months of age, VAS had a beneficial effect on non-rotavirus diarrhoea [32] and was also associated with less measles hospitalisations and deaths [33]. Given such wide ranging immunological effects and the uncertainty about whether NNVAS is beneficial or not, this study set out to investigate the effect of NNVAS in a West African neonatal population.

## Methods/design

### Study design

This trial was designed to provide indicative data on the immunological impact of NNVAS to infants born in a peri-urban area of The Gambia; an area of moderate VAD. Two hundred mother–infant pairs were enrolled in a single centre, phase II, double-blind, RCT. Mother-infant pairs were approached shortly after delivery, with randomisation occurring within 48 hours of birth. A range of immunological outcomes were examined up until the 17^th^ week of life and participation continued for one year to ensure that all Expanded Programme of Immunisation (EPI) vaccines were administered including 100,000 IU VA to infants at 6 months of age and 200,000 IU VA at 12 months. During this additional follow-up period, morbidity and anthropometry data were collected. Recruitment commenced in December 2011 and was completed in October 2012. The last study participant reached the 17^th^ week of life in February 2013 and graduated from the trial in October 2013. All field and laboratory staff remained blinded throughout the trial. Following database lock of the ‘immunology phase’ of the trial (data up to the 17^th^ week of life), the principal investigator (PI) was partially unblinded to group level only; (not placebo/intervention assignment). Data analysis is still ongoing.

### Participants and study setting

Mother-infant pairs (n = 200) were recruited at the Sukuta Health Centre; a government health clinic, in the Western coastal region of The Gambia. The health centre has a peri-urban catchment area ie: surrounding an urban town with characteristics of a rural setting; characterised by low income and crowded living conditions. The Gambia has a population of 1.8 million of which 57% reside in the urbanised coastal area [34]. There is a low adult HIV prevalence (1.5%)[35] and an infant mortality rate of approximately 66.4 per 1,000 live births [34]. Data from previous studies indicate a high prevalence of VAD among women and children. In 2001 the Gambian National Nutrition Agency (NaNA) conducted a nationwide survey of VAD, which demonstrated over 70% moderate VAD (serum retinol < 0.70 μmol/L) in pregnant and lactating mothers, infants and under 5 years in most areas, including coastal regions [36]. More recently, an RCT that examined the safety and efficacy of early high-dose VAS in 220 rural Gambian infants indicated a prevalence of 58% of VAD in cord blood [37].

The local community was sensitized to the study through an open day organised at MRC Sukuta clinic and through field workers known to the community. Attendance at government antenatal clinics in Sukuta varies greatly and is unregulated; however pictorial representation of the trials information sheet was displayed at the antenatal clinic. Mothers delivering at the Government health centre were approached shortly after delivery by a trained MRC nurse or field worker. Those that were interested in participating in the trial had all details of the study explained to them in their local language, covering all aspects of the trial, as laid out in the information sheet. Any questions that arose were answered by the nurse or field worker, or referred to the PI for clarification. Subjects were also offered the opportunity to speak to the PI or study clinician if they wished. If the subject agreed to participate, a short assessment of understanding was conducted, which consisted of 8 true/false questions about what participating in the trial entailed. Mothers who answered all questions correctly were invited to complete the informed consent form (ICF). Mothers that had a maximum of 2 incorrect answers had the information sheet re-explained to them. At the second attempt, if all questions were answered correctly, mothers were invited to complete the ICF. Mothers that were unable to answer all the questions correctly at the second attempt were not invited to complete the ICF. Depending on the level of literacy, written informed consent was obtained through either a signature or thumb print. In the case of illiterate mothers, an impartial witness (government nurse) was present throughout the consenting process and counter signed the informed consent form.

Throughout the trial, all participants had access to free clinical consultations at the Sukuta MRC clinic; essential drugs were provided free of charge and if required, they had access to the clinical services available at the main MRC unit at the coast.

### Trial administration and oversight

This trial was approved by the Joint Gambia Government/Medical Research Council ethics committee (local ethics committee (LEC); project number SCC1198), the London School of Hygiene and Tropical Medicine (LSHTM) ethics committee (application number A277 5697) and the WHO ethics review committee (protocol ID RPC389). Approval from The Republic of The Gambia Government Ministry of Health and Social Welfare and The Republic of The Gambia National Pharmaceutical Services Medicine Board was granted prior to participant recruitment. The trial was periodically monitored by the clinical trials support office (CTSO), MRC Gambia unit, with a primary role of monitoring adherence to the trial protocol and ICH-GCP guidelines. In addition, quality assurance audits were conducted by both the quality department, (MRC Gambia unit) and the sponsor’s quality assurance manager (LSHTM) to ensure adherence to the trial protocol and ICH-GCP guidelines. A detailed structured review of study implementation was conducted by WHO technical staff on a site visit shortly after recruitment commenced. The local safety monitor (LSM) (consultant paediatrician, clinical services department, MRC Gambia unit) had the primary role of independently monitoring all serious adverse events (SAEs) (as defined by ICH-GCP guidelines) and all adverse events (AEs) occurring within 72hs of supplementation. The data safety monitoring board (DSMB) was sponsored by WHO and monitored both adherence to the trial protocol and supervised the progress of the trial toward its objectives via interim analyses of selected outcome data by randomization groups. The DSMB met a total of five times and conducted interim analyses of the safety data, pertaining to the occurrence of AE/SAEs. In addition, reports on SAEs were submitted to the DSMB, designated WHO officials, LSHTM quality assurance manager, CTSO and LSM in real time. Stopping criteria for the trial were as follows: (a) Increased mortality in one group compared to the other defined as either (i) mortality 2 times higher in one group compared to the other, (ii) rate exceeding 6 neonatal deaths per 100 subjects in either group, (iii) rate exceeding 11 infant deaths per 100 subjects in either group); (b). Preponderance of evidence of other side effects; (c) Overwhelming evidence of early benefit in one group compared to the other.

### Inclusion criteria and exclusion criteria

Inclusion criteria included singleton birth, birth weigh ≥ 1,500 g, mothers over 18 years, residency within the study area and administration of birth vaccinations and VAS within 48hs of birth. Exclusion criteria were infants having a congenital disease, a serious infection at birth or an inability to feed (initially assessed by the lack of a suck reflex), mothers who were seriously ill at the time of enrolment (defined as bed bound for more than 24 hs), mother participating in other studies and/or mothers who were known to be HIV infected. In cases where HIV status of the mother was not known at birth, a subsequent positive test result at 1 week post-partum led to exclusion before the six week time point (time of first bleed).

### Randomisation and allocation

Randomization occurred within 48 hs of birth, once it has been established that the neonate met all inclusion criteria and that the mother had provided informed consent. Randomization accommodated stratification by sex and mean birth weight (over/under 3,150 g), to allow for later analyses by sex and to allow enrolment of infants with a range of nutritional status. The randomization lists was generated by WHO, using Stata, v11 (http://www.stata.com/products/stb/journals/stb41.pdf, command “ralloc”, p.44). Randomization was performed in blocks of four to ensure a balance in sample size across groups over time. This compensated for any potential seasonal effects on vaccine and VA responses. Each neonate enrolled into the trial was given the next available supplement from the box of supplements that corresponded to their birth weight and sex grouping (eg: female above 3,150 g; males below 3,150 g *etc.*).

### Intervention

The VA capsules (soybean oil carrier with 50,000 IU retinyl palmitate and minute quantities of vitamin E) and placebo (soybean oil without VA) were prepared by Strides Arcolab Limited, (Bangalore, India). These capsules are from the same batches prepared for the NNVAS mortality trials [11]. Capsules and corresponding packaging (photosensitive blister packs containing 2 identical capsules) appeared identical for intervention and placebo. Within each blister pack, one capsule was used for administration to the neonate, whilst the other was used as either a back-up capsule if the first dose was accidentally spilled, or for randomly selected stability testing (conducted periodically throughout the trial by an independent laboratory (VITAS, Norway)). Blinded codes were assigned to each blister pack by a designated WHO official in Geneva, with the code held securely by the WHO official and two additional independent custodians. Supplements were securely stored in an air conditioned room (24°C) with the temperature recoded daily. On each designated supplementation day, four boxes of supplements were transported to the clinic in a cool box containing chilled cool packs, (to ensure temperatures did not reach in excess of 30°C whilst in the field) with temperatures recorded periodically. Oral supplementation was conducted by one of two trained study staff and supervised by either the PI, or a designated supervisor. The supplement was administered by cutting the tapered end of the capsule with scissors and squeezing the complete contents of the capsule directly into the neonate’s mouth.

### Study follow-up period

**Figure 1:**
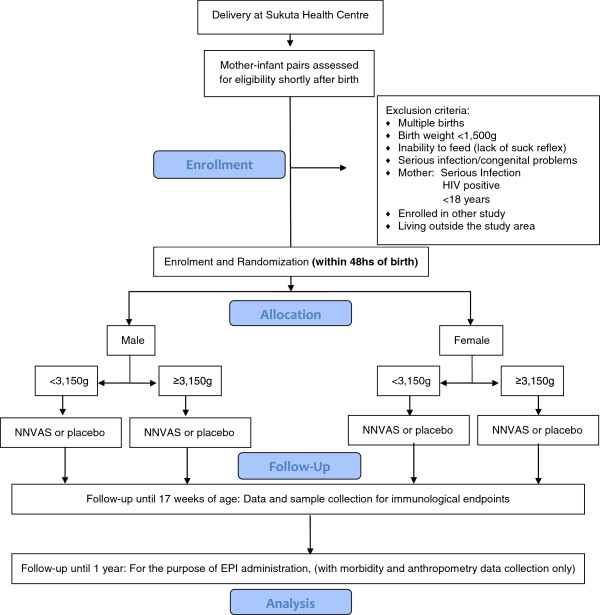
**Flow chart of the study design.** Key: EPI; expanded programme of immunisation; NNVAS; neonatal vitamin A supplementation.

All infants were followed-up from birth to one year of age and received their childhood vaccines according to the recommended EPI schedule in The Gambia (Table 1). The flow chart of the study is presented in Figure 1. For each infant, two adverse event home visits were carried out by trained nurses at 24 and 72 hs post-supplementation. Expected adverse events were bulging fontanelle, diarrhoea, vomiting, fever, inability to suck or feed and convulsions. In cases were infants were unwell, vital signs were recorded and the infant was immediately brought to the MRC Sukuta clinic for review by the trial physician (or MRC hospital ward staff if the trial physician was unavailable).

**Table 1:** EPI Schedule in The Gambia During the First Year of Life

**Age (week)**	**Vaccine**
0 (within 48 hours of birth)	BCG, HBV, OPV
8	Pentavalent, OPV, PCV-13
12	Pentavalent, OPV, PCV-13
16	Pentavalent, OPV, PCV-13
26	VAS (100,000 IU)
40	Measles, Yellow Fever, OPV
52	VAS (200,000 IU)

Key: BCG; bacillus Calmette-Guerin, OPV; oral poliovirus vaccine, HBV; hepatitis B vaccine, Pentavalent (DTwP, Hib, HBV (DTwP: diptheria, tetanus, whole cell pertussis; Hib: *Haemophilus Influenza* type b)), PCV-13; 13-valent pneumococcal conjugate vaccine, VAS; vitamin A supplementation.

### Measurements and sample collection

Following delivery, infants were seen at the MRC Sukuta clinic within 48hs of birth and at 1, 6, 8, 12, 16 and 17 weeks of age. Home visits were conducted for the two post-supplementation AE visits, at three weeks of age (for morbidity assessment) and at six weeks of age (+ 72 hs) for tuberculin skin test (TST) readings. In a subset of 53 participants a modified relative dose response (MRDR) test was administered at home at 17 weeks of age (prior to the 17 week clinic visit). This test has been described previously [38] and is an accurate indicator of VA liver stores. Table 2 details the data and sample collection schedule for each visit. At all time points infant anthropometric measurements were made using standard protocols with regularly validated equipment, including weight, length, head circumference and mid-upper arm circumference (MUAC). At all trial visits, morbidity (including vital signs) and breastfeeding practices since the previous visit were assessed. Maternal anthropometric measurements were taken after delivery and at 6, 12 and 17 weeks. A demographic and socio-economic questionnaire was completed at the one week visit, along with questionnaires regarding the mothers feeding practices seven days prior to delivery and her medical history during pregnancy. Venous blood samples were collected from the mothers at the one week visit (for HIV testing) and at six weeks (to assess serum retinol status). Following their six week blood draw, all mothers received 200,000 IU VAS as per Gambian Government post-partum protocol. It was ensured that the supplement was not given to the mothers prior to their six week blood draw.

**Table 2:** Sample and data collection time points

	**Infant age (weeks)**										
**Measurement**	0*	**1**	**3**	**6**	**8**	**12**	**16**	**17**	**26**	**40**	**52**
**EPI**	X				X	X	X		X	X	X
**Thymic index**		X		X		X		X			
**Venous bleed (Infant)**				X				X			
**Venous bleed (Mothers)**		X		X							
**Tuburculin skin test**				X							
**Modified relative dose response**								X			
**Anthropometry (Infant)**	X	X		X	X	X	X	X	X	X	X
**Anthropometry (Mother)**	X			X		X		X			
**Morbidity**	X	X	X	X	X	X	X	X	X	X	X

*within 48hs of birth Note: Adverse event visits were carried out at 24 and 72 hours post-supplementation. Mothers’ venous bleed at 1 week was to determine HIV status before the 6 week time point.

Trained study personnel administered EPI vaccines to all infants, as per Gambian Government protocol (Table 1). All study vaccines which were administered during the immunological phase of the trial (first 17 weeks of life) were acquired directly from the designated UNICEF manufacturers with cold-chain managed thereafter by the study team. For vaccination during the additional ‘EPI follow-up’ phase of the trial (6, 9 and 12 months age), vaccines and supplements were acquired from the EPI Department of the Gambia Government, and the cold-chain managed thereafter by the local government health clinic. At 6 and 17 weeks of age, a venous blood sample was collected from infants for full blood count, *ex vivo* flowcytometric analysis of B cells, T_reg_ cells, DCs and T cells, anti-hepatitis B antibody titre (17 week sample only), MRDR test (subset of 17 week samples only), serum retinol status and inflammatory markers. Residual aliquots were banked for future analysis.

Neonatal/infant thymus size was assessed sonographically at 1, 6, 12 and 17 weeks using a validated method in which the transverse diameter of the thymus and the saggital area of its largest lobe are multiplied to give a volume-related thymic index (TI) [39]. In addition, DNA extracted from peripheral blood mononuclear cells at the 17 week time point were used for T cell receptor excision circles (TRECs) analysis as a further marker of thymic function [40].

### Trial outcomes

The primary endpoint of this trial is the frequency of circulating T_reg_ cells expressing gut homing receptors in infants at 17 week post-supplementation; the null hypothesis being that NNVAS will increase the frequency of gut homing T_reg_ cells compared to the control arm. Secondary analysis will compare the following variables between trial arms; thymus maturation (assessed at 1, 6, 12 and 17 weeks using TI and TRECs analysis at 17 weeks) and enhanced B cell immune responses, (assessed via *ex vivo* flow cytometric analysis at 6 and 17 weeks (frequency and cell subtype) along with Hepatitis B antibody responses at 17 weeks post-supplementation). In addition to the primary and secondary outcomes listed on *clinicaltrials.gov*, the following hypotheses were investigated: i) NNVAS diminishes the TST response; ii) NNVAS decreases inflammatory markers. Sample collection has been completed to address the following hypotheses too, however due to funding restrictions data are still pending; i) NNVAS skews mycobacterial and recall antigen responses towards a Th2 profile; ii) NNVAS diminishes Th1 and Th17 reactivity to mycobacterial and recall antigens; iii) NNVAS causes increased innate immune reactivity; iv) NNVAS increases circulating immunoglobulin A (IgA) in the mucosal immune compartment, especially oral polio vaccine (OPV) specific IgA post-vaccination; v) NNVAS decreases bacterial translocation, by improving mucosal barrier function.

### Analysis plan

All baseline data will be described using appropriate summary statistics i.e. mean and standard deviation for normally distributed variables and median and inter-quartile range for non-normally distributed variables.

Data also of interest, although not part of the primary or secondary analysis, are the comparison between trial arms of serum retinol levels (indirectly measured via retinol binding protein), MRDR values, anthropometry data (of both infant and mother), morbidity and mortality, mother feeding practices prior to delivery, infant feeding practices, socio-economic status, demographic data and pregnancy medical history. *Ex vivo* flow cytometric analysis of DCs and T cell panels was also conducted. For the primary and secondary analysis both linear regression (for continuous outcomes such as T_reg_ cells) and logistic regression (for binary outcomes such as TST response) will be used to quantify the difference between trial arms. The regression models, where necessary, will allow for repeated measures within individuals over time using random effects and all relevant covariates will be taken into account. Model assumptions will be checked to confirm reliable estimates and appropriate transformations applied to the outcome variable. The randomization is stratified by birth weight and sex and so both will be taken into account for all analysis. Significance is defined as p < 0.05. All analysis will be performed in Stata v12.1 (StataCorp LP, USA, http://www.stata.com).

### Sample size

A sample of size of 200 was based on previous similar immunological studies carried out in the same cohort[41], alongside logistical and financial factors. Knowledge on the potential immunological sequelae of human NNVAS is so scarce that the foremost aim of this study is to seek indicative data on aspects of immunity likely to be affected by NNVAS which can then be subjected to adequately powered investigations where power can be calculated based on variances derived in this study (and its Bangladeshi counterpart).

## Discussion

The trials in The Gambia and Bangladesh will shed light on the potential biological mechanisms by which NNVAS modulates the human neonatal immune system. With the animal metabolism study which has been run in parallel, these three studies were designed to complement the large mortality trials[11] which alongside existing data from previous NNVAS trials (conducted in Indonesia, Bangladesh, Nepal, India, Guinea-Bissau and Zimbabwe[5-9,42]) will provide definitive information in formulating global policy for this intervention.

### Trial status

Ongoing; recruitment and follow-up is completed, partially unblinded analysis is still ongoing.

## Abbreviations

AE: Adverse event; APC: Antigen presenting cell; BCG: Bacillus Calmette-Guerin; CTSO: Clinical trial support office; DC: Dendritic cells; DSMB: Data safety monitoring board; DTP: Diptheria Tetanus Pertussis; EPI: Expanded programme of immunisation; Hs: Hours; HBV: Hepatitis B vaccine; Hib: *Haemophilus Influenza* Type b; HIV: Human immunodeficiency virus; ICF: Informed consent form; ICH-GCP: International conference on harmonization-Good clinical practice; IgA: Immunoglobulin A; IU: International units; LEC: Local ethics committee; LSHTM: London School of Hygiene and Tropical Medicine; LSM: Local safety monitor; MRC: Medical Research Council; MRDR: Modified relative dose response; MUAC: Mid-upper arm circumference; NNVAS: neonatal vitamin A supplementation; OPV: Oral Polio vaccine; PCV-13: Pneumococcal conjugate vaccine-13; PI: Principal investigator; RA: Retinoic acid; RCT: Randomised controlled trial; SAE: Serious adverse event; Th: Helper T cell; TI: Thymic index; TRECs: T cell receptor excision circles; Treg: T regulatory cells; TST: Tuberculin skin test; VA: vitamin A; VAD: Vitamin A deficiency; VAS: Vitamin A supplementation.

## Competing interests

The authors declare that they have no competing interests.

## Authors’ contributions

SLRM and AMP are the principal investigators. MS, KLF, AJCF, AMP and SLRM all contributed to the development of the trial protocol. AJCF and SLRM drafted the statistical analysis plan and LK assisted SLRM with the finalisation. SLRM drafted this manuscript and all authors reviewed critically for content and approved the final manuscript. All authors read and approved the final manuscript.

## Pre-publication history

The pre-publication history for this paper can be accessed here:

http://www.biomedcentral.com/1471-2431/14/92/prepub
